# SNP-SNP interaction analysis of NF-κB signaling pathway on breast cancer survival

**DOI:** 10.18632/oncotarget.4991

**Published:** 2015-07-22

**Authors:** Maral Jamshidi, Rainer Fagerholm, Sofia Khan, Kristiina Aittomäki, Kamila Czene, Hatef Darabi, Jingmei Li, Irene L. Andrulis, Jenny Chang-Claude, Peter Devilee, Peter A. Fasching, Kyriaki Michailidou, Manjeet K. Bolla, Joe Dennis, Qin Wang, Qi Guo, Valerie Rhenius, Sten Cornelissen, Anja Rudolph, Julia A. Knight, Christian R. Loehberg, Barbara Burwinkel, Frederik Marme, John L. Hopper, Melissa C. Southey, Stig E. Bojesen, Henrik Flyger, Hermann Brenner, Bernd Holleczek, Sara Margolin, Arto Mannermaa, Veli-Matti Kosma, kConFab Investigators, Laurien Van Dyck, Ines Nevelsteen, Fergus J. Couch, Janet E. Olson, Graham G. Giles, Catriona McLean, Christopher A. Haiman, Brian E. Henderson, Robert Winqvist, Katri Pylkäs, Rob A.E.M. Tollenaar, Montserrat García-Closas, Jonine Figueroa, Maartje J. Hooning, John W.M. Martens, Angela Cox, Simon S. Cross, Jacques Simard, Alison M. Dunning, Douglas F. Easton, Paul D.P. Pharoah, Per Hall, Carl Blomqvist, Marjanka K. Schmidt, Heli Nevanlinna

**Affiliations:** ^1^ Department of Obstetrics and Gynecology, University of Helsinki and Helsinki University Hospital, Helsinki, FI-00029 HUS, Finland; ^2^ Department of Clinical Genetics, University of Helsinki and Helsinki University Hospital, Helsinki, FI-00029 HUS, Finland; ^3^ Department of Medical Epidemiology and Biostatistics, Karolinska Institutet, Stockholm SE-17177, Sweden; ^4^ Lunenfeld-Tanenbaum Research Institute of Mount Sinai Hospital, Toronto, ON, M5G 1X5, Canada; ^5^ Department of Molecular Genetics, University of Toronto, Toronto, ON, Canada; ^6^ Department of Obstetrics and Gynecology, University of Ulm, Ulm, Germany; ^7^ Division of Cancer Epidemiology, German Cancer Research Center (DKFZ), Heidelberg, Germany; ^8^ Department of Pathology, Leiden University Medical Center, Leiden, The Netherlands; ^9^ Department of Human Genetics, Leiden University Medical Center, Leiden, The Netherlands; ^10^ Department of Gynecology and Obstetrics, University Hospital Erlangen, Friedrich-Alexander University Erlangen-Nuremberg, Erlangen, Germany; ^11^ Department of Medicine, Division of Hematology and Oncology, University of California at Los Angeles, Los Angeles, CA, USA; ^12^ Centre for Cancer Genetic Epidemiology, Department of Public Health and Primary Care, University of Cambridge, Cambridge, UK; ^13^ Centre for Cancer Genetic Epidemiology, Department of Oncology, University of Cambridge, Cambridge, UK; ^14^ Netherlands Cancer Institute, Antoni van Leeuwenhoek hospital, Amsterdam, The Netherlands; ^15^ Prosserman Centre for Health Research, Lunenfeld-Tanenbaum Research Institute of Mount Sinai Hospital, Toronto, ON, M5G 1X5, Canada; ^16^ Division of Epidemiology, Dalla Lana School of Public Health, University of Toronto, Toronto, ON, M5S 1A8, Canada; ^17^ Department of Gynaecology and Obstetrics, University Hospital Erlangen, Friedrich-Alexander University Erlangen-Nuremberg, Comprehensive Cancer Center Erlangen-EMN, Erlangen, Germany; ^18^ Molecular Epidemiology Group, German Cancer Research Center, Heidelberg, Germany; ^19^ Department of Obstetrics and Gynecology, University of Heidelberg, Heidelberg, Germany; ^20^ National Center for Tumor Diseases, University of Heidelberg, Heidelberg, Germany; ^21^ Centre for Epidemiology and Biostatistics, Melbourne School of Population and Global Health, The University of Melbourne, Melbourne, Victoria, Australia; ^22^ Department of Pathology, The University of Melbourne, Melbourne, Victoria, Australia; ^23^ Faculty of Health and Medical Sciences, University of Copenhagen, Copenhagen, Denmark; ^24^ Department of Clinical Biochemistry, Herlev Hospital, Copenhagen University Hospital, Herlev, Denmark; ^25^ Department of Breast Surgery, Herlev Hospital, Copenhagen University Hospital, Herlev, Denmark; ^26^ Division of Clinical Epidemiology and Aging Research, German Cancer Research Center (DKFZ), Heidelberg, Germany; ^27^ Division of Preventive Oncology, German Cancer Research Center (DKFZ) and National Center for Tumor Diseases (NCT), Heidelberg, Germany; ^28^ German Cancer Consortium (DKTK), German Cancer Research Center (DKFZ), Heidelberg, Germany; ^29^ Saarland Cancer Registry, Saarbrücken, Germany; ^30^ Department of Oncology - Pathology, Karolinska Institutet, Stockholm, Sweden; ^31^ School of Medicine, Institute of Clinical Medicine, Pathology and Forensic Medicine, University of Eastern Finland, Kuopio, Finland; ^32^ Cancer Center of Eastern Finland, University of Eastern Finland, Kuopio, Finland; ^33^ Imaging Center, Department of Clinical Pathology, Kuopio University Hospital, Kuopio, Finland; ^34^ Peter MacCallum Cancer Center, Melbourne, Victoria, Australia; ^35^ Vesalius Research Center (VRC), VIB, Leuven, Belgium; ^36^ Laboratory for Translational Genetics, Department of Oncology, University of Leuven, Leuven, Belgium; ^37^ Multidisciplinary Breast Center, Medical Oncology, University Hospital Leuven, Leuven, Belgium; ^38^ Department of Laboratory Medicine and Pathology, Mayo Clinic, Rochester, MN, USA; ^39^ Department of Health Sciences Research, Mayo Clinic, Rochester, MN, USA; ^40^ Cancer Epidemiology Centre, Cancer Council Victoria, Melbourne, Australia; ^41^ Centre for Epidemiology and Biostatistics, School of Population and Global health, The University of Melbourne, Melbourne, Australia; ^42^ Anatomical Pathology, The Alfred Hospital, Melbourne, Australia; ^43^ Department of Preventive Medicine, Keck School of Medicine, University of Southern California, Los Angeles, CA, USA; ^44^ Laboratory of Cancer Genetics and Tumor Biology, Cancer Research and Translational Medicine, Biocenter Oulu, University of Oulu, Oulu, Finland; ^45^ Laboratory of Cancer Genetics and Tumor Biology, Northern Finland Laboratory Centre NordLab, Oulu, Finland; ^46^ Department of Surgical Oncology, Leiden University Medical Center, Leiden, The Netherlands; ^47^ Division of Genetics and Epidemiology, Institute of Cancer Research, Sutton, SM2 5NG, UK; ^48^ Breakthrough Breast Cancer Research Centre, Division of Breast Cancer Research, The Institute of Cancer Research, London, SW3 6JB, UK; ^49^ Division of Cancer Epidemiology and Genetics, National Cancer Institute, Rockville, MD, USA; ^50^ Department of Medical Oncology, Erasmus MC Cancer Institute, AE Rotterdam, The Netherlands; ^51^ Sheffield Cancer Research, Department of Oncology, University of Sheffield, Sheffield, UK; ^52^ Academic Unit of Pathology, Department of Neuroscience, University of Sheffield, Sheffield, UK; ^53^ Centre Hospitalier Universitaire de Québec Research Center, Laval University, Québec City, Canada; ^54^ Department of Oncology, University of Helsinki and Helsinki University Central Hospital, Helsinki, HUS, Finland

**Keywords:** breast cancer, survival analysis, SNP-SNP interaction, NF-κB pathway

## Abstract

In breast cancer, constitutive activation of NF-κB has been reported, however, the impact of genetic variation of the pathway on patient prognosis has been little studied. Furthermore, a combination of genetic variants, rather than single polymorphisms, may affect disease prognosis. Here, in an extensive dataset (*n* = 30,431) from the Breast Cancer Association Consortium, we investigated the association of 917 SNPs in 75 genes in the NF-κB pathway with breast cancer prognosis. We explored SNP-SNP interactions on survival using the likelihood-ratio test comparing multivariate Cox’ regression models of SNP pairs without and with an interaction term. We found two interacting pairs associating with prognosis: patients simultaneously homozygous for the rare alleles of rs5996080 and rs7973914 had worse survival (HR_interaction_ 6.98, 95% CI=3.3-14.4, *P* = 1.42E-07), and patients carrying at least one rare allele for rs17243893 and rs57890595 had better survival (HR_interaction_ 0.51, 95% CI=0.3-0.6, *P* = 2.19E-05). Based on in silico functional analyses and literature, we speculate that the rs5996080 and rs7973914 loci may affect the BAFFR and TNFR1/TNFR3 receptors and breast cancer survival, possibly by disturbing both the canonical and non-canonical NF-κB pathways or their dynamics, whereas, rs17243893-rs57890595 interaction on survival may be mediated through TRAF2-TRAIL-R4 interplay. These results warrant further validation and functional analyses.

## INTRODUCTION

Aberrant regulation of the NF-κB is common in human breast cancer cell lines and in primary tumor cells from patients with breast cancer [1, 2]. Dysregulation of the NF-κB signaling pathway has been shown to contribute to cancer development and progression as well as to chemo-and radiotherapy-resistance [3], and selective inhibition of the NF-κB-activating pathway genes has been reported to sensitize breast cancer cell lines to doxorubicin [2, 4-6]. To date, few studies have investigated whether inherited genetic variation in the NF-κB pathway is associated with breast cancer prognosis [7, 8]. Our previous research indicates a link between breast cancer outcome and the *NQO1* gene, the expression and function of which is closely connected to the NF-κB network [9-12]. Recently, a breast cancer survival study on SNPs within or in the 100kb flanking region of genes implicated in human immunology and inflammation suggested that rs4458204 affects breast cancer survival in patients with ER-negative tumors who have been treated with chemotherapy. Rs4458204 is located 41.5 kb upstream of the chemokine ligand 20 (CCL20) which is a downstream target of NF-κB [13].

Two sub-pathways are proposed to account for the NF-κB activation: the canonical, and the non-canonical NF-κB pathway. The canonical pathway, also referred to as the classic pathway, mainly activates the NF-κB1 dimers (RelA:p50). Under resting conditions, the NF-κB1 dimers are bound to IκB-α and retained in an inactive form in the cytoplasm. When the canonical pathway is stimulated by pro-inflammatory signals (e.g. TNFα) through their receptors (e.g.TNFR1) the signal is transduced through a FADD and TRAF2 assembly to the IKKβ-NEMO complex which phosphorylates the IκB molecules, and this in turn leads to degradation of IκB-α. Upon IκB-α degradation, the NF-κB1 dimers are free to translocate to the nucleus where they activate target gene transcription. The canonical pathway therefore consists of the ligands, the receptors, the IKK complex, IκB proteins and NF-κB dimers which together can lead to either cell survival or cell death [14-17].

The non-canonical pathway involves the activation of IKKα by the TNF cytokines (e.g. BAFF and LT-β through their receptors (e.g. BAFFR and TNFR3, also known as LT-βR). This results in the phosphorylation and degradation of p100, and the subsequent formation of the p52 (processed NF-κB2) and RelB complex. The p52-RelB complex then translocates to the nucleus and induces the expression of a different set of target genes than those induced by the canonical pathway [18, 19]. Some overlap exists between the canonical and non-canonical pathways: signals associated with cell survival or apoptosis can be transmitted via the same ligand and/or receptor in both pathways [20].

Here, we investigated the association between breast cancer patient survival and SNPs residing within or in the 50-kb flanking region of 75 genes in the NF-κB activating pathway using an extensive data set of the Breast Cancer Association Consortium (BCAC). It is plausible that a combination of genetic variants, rather than a single polymorphism, may affect the prognosis for a complex disease such as breast cancer. For instance, a recent combinatorial RNAi screening of cancer genes, which are frequently co-altered in primary breast cancer, identified interacting gene pairs that associate with patient survival [21]. Here, in addition to a single SNP association study, we present two-SNP interaction analysis, which is more feasible to conduct and interpret than multiple-SNP interactions [22]. The panel of markers included 917 SNPs for 75 candidate genes involved in the activation of the NF-κB pathway available in a custom Illumina iSelect genotyping array designed for the Collaborative Oncological Gene-Environment Study (iCOGS) [23].

## RESULTS

### SNP association with prognosis

Altogether 30,431 invasive breast cancer cases of European ancestry from 24 BCAC studies participated in this study (Supplementary Table 1) [23]. A total of 917 SNPs available in the BCAC iCOGS data set (See material and methods) were included in the analyses to investigate the association of the SNPs and breast cancer patient survival. All SNPs were located within, or in a 50kb flanking region of 75 candidate genes involved in the activation of the NF-κB pathway. All survival analyses were adjusted for study. Ten-year overall survival (death due to breast cancer or other reasons) was used as the end-point in these analyses for reasons of data availability and consistency. The survival analyses of the 917 single SNPs studied here revealed no significant (*P*_Corrected_ > 0.01) association with patient survival (Supplementary Table 2). To discover plausible epistatic/non-additive interacting SNPs associating with prognosis, we conducted a two-SNP interaction survival analysis using the likelihood ratio test comparing multivariate Cox’ regression models of pairs of SNPs without and with an interaction term (SNP1+SNP2 *vs*. SNP1+SNP2+(SNP1*SNP2), respectively) (see methods/statistical analyses). Using Cox proportional hazard models, the recessive (AA = 0, Aa = 0, aa = 1) and dominant (AA = 0, Aa = 1, aa = 1) models were assessed for all the SNPs. With a sample size of 30,431 with 3375 events and the average MAF of 23.4%, we had 80% power to detect interaction terms with HR above 1.4 (or 1/1.4 = 0.6) and HR of 6.2 (or 1/6.2 = 0.16) in the dominant and recessive models respectively. We found one interacting SNP pair, rs5996080 and rs7973914, in the recessive model and another one, rs17243893 and rs57890595, in the dominant model which passed these HR thresholds with a corrected p-value threshold <0.05.

**Figure 1:**
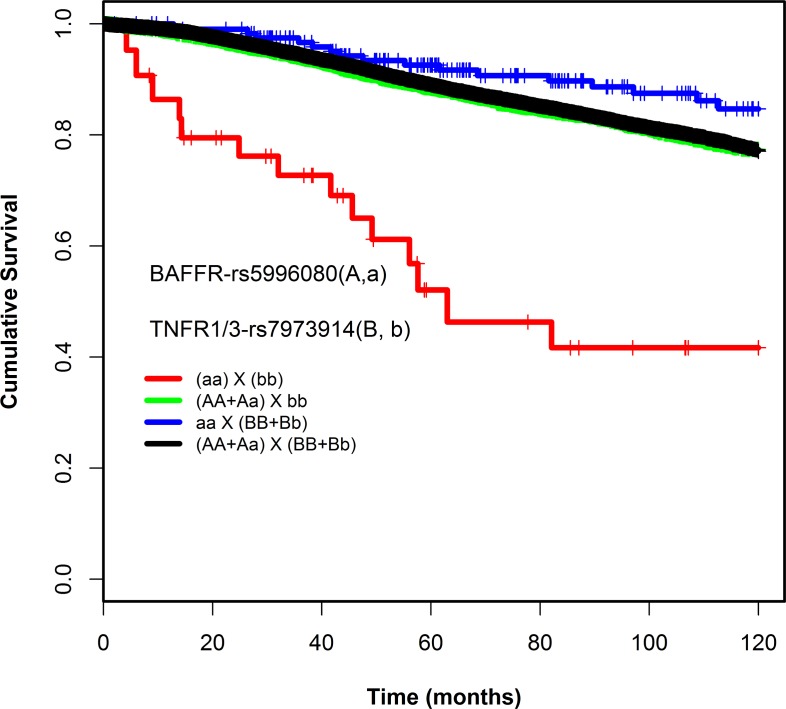
Kaplan-Meier survival curves of the combination genotypes of rs5996080 and rs7973914 (recessive model)

Under the recessive model, a pair-wise interaction between rs5996080 (A/G, MAF = 8%) and rs7973914 (G/A, MAF = 40%) was found to be associated with patient survival: patients carrying the homozygous rare allele for both SNPs (rs5996080-GG, rs7973914-AA) had worse overall survival compared to carriers of at least one common allele (HR_interaction_ 6.98, 95% CI = 3.3-14.4, *P =* 1.42E-07, Table 1). The interaction was statistically significant in a likelihood-ratio test, compared to a Cox model without an interaction term (*P*_likelihood-ratio-corrected_ = 0.003) (Table 1). Absolute uncorrected survival rates of genotype combination categories were compared using Kaplan-Meier curves (Figure 1). Supplementary table 3 lists the likelihood-ratio test p values obtained also for the SNPs nearby or in LD with the rs5996080-rs7973914 interacting pair. Due to the limited power (small number of patients and less than 5 events per subgroup), no subgroup analyses by ER status, lymph node status, and chemotherapy treatment (see methods) were conducted for this interacting pair.

**Table 1:** Multivariate Cox' regression models to assess the interaction between rs5996080 and rs7973914 by recessive model of inheritance

**Model without an interaction term**				
	**n(death)**	**HR**	**95% CI**	**P**
rs5996080 (A, a)				
AA+Aa	30173 (3345)	1 (Ref.)		
aa	258 (30)	1.07	(0.7-1.5)	0.695
rs7973914 (B, b)				
BB+Bb	25321 (2800)	1 (Ref.)		
bb	5106 (575)	1.03	(0.9-1.1)	0.461
**Model with an interaction term(aa X bb)**				
	**n(death)**	**HR**	**95% CI**	**P**
rs5996080 (A, a)				
AA+Aa		1 (Ref.)		
aa		0.64	(0.4-1.0)	0.079
rs7973914 (B, b)				
BB+Bb		1 (Ref.)		
bb		1.01	(0.9-1.1)	0.809
**Interaction**				
aa X bb	46(14)	6.98	(3.3-14.4)	1.42E-07
Liklihood ratio test between the models*				
				**P_corrected_**
				**0.003**

All the models are adjusted for study

* likelihood ratio test comparing Cox' regression model without and with an interaction term.

**Figure 2:**
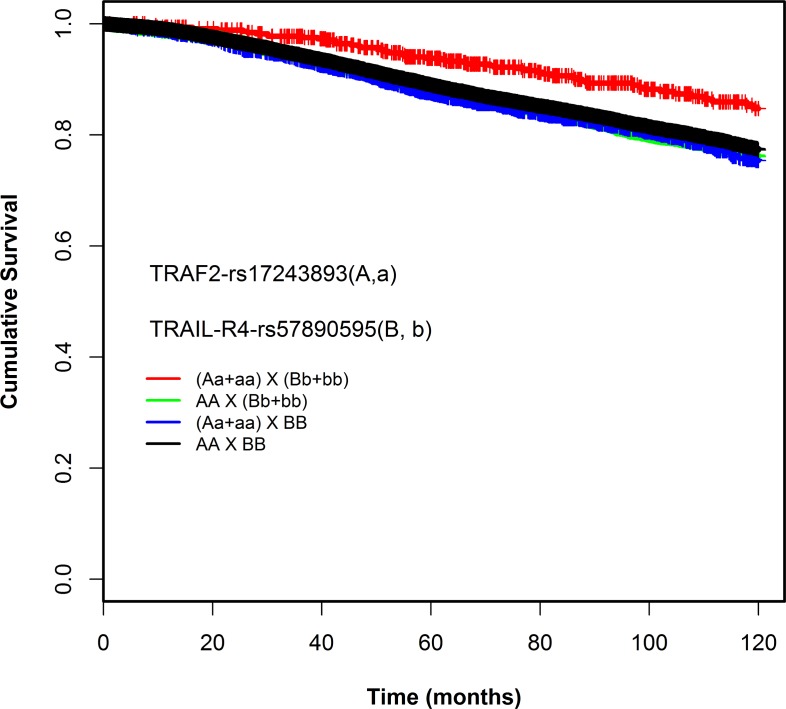
Kaplan-Meier survival curves of the combination genotypes of rs17243893 and rs57890595 (dominant model)

Using the dominant model, we found a pair-wise interaction between rs17243893 (A/G, MAF = 5%) and rs57890595 (A/C, MAF = 11%) to be associated with patient survival: Patients simultaneously carrying at least one rare allele for both variants (rs17243893-AG+GG, rs57890595-AC+CC) had better overall survival compared to the patients with common homozygous genotypes (HR_interaction_ 0.51, 95% CI = 0.3-0.6, *P =* 2.19E-05) (Table 2). The interaction was statistically significant (*P*_likelihood-ratio-corrected_ = 0.005) (Table 2). Absolute uncorrected survival rates of genotype combination categories were compared using Kaplan-Meier curves (Figure 2). Supplementary table 4 lists the likelihood-ratio test p values obtained also for the SNPs nearby or in LD with the rs17243893-rs57890595 interacting pair. The effect of the SNP-SNP interaction on patient survival was consistent between the subgroups studied (ER positive *vs*. negative, lymph node positive *vs*. negative, chemotherapy-treated *vs*. non-treated patients) and was not confined to any specific subset of patients (Supplementary Table 5, Supplementary Figure 1).

**Table 2:** Multivariate Cox' regression models to assess the interaction between rs17243893 and rs57890595 by dominant model of inheritance

**Model without an interaction term**				
	**n (death)**	**HR**	**95% CI**	**P**
rs17243893 (A, a)				
AA	26994 (3018)	1 (Ref.)		
Aa+aa	3096 (334)	0.95	(0.8-1.0)	0.464
rs57890595				
BB	23393 (2590)	1 (Ref.)		
Bb+bb	6781 (756)	1.01	(0.9-1.0)	0.87
**Model with an interaction term ((Aa+aa) X (Bb+bb))**				
	**n (death)**	**HR**	**95% CI**	**P**
rs17243893 (A, a)				
AA		1 (Ref.)		
Aa+aa		1.09	(0.9-1.2)	0.139
rs57890595				
BB		1 (Ref.)		
Bb+bb		1.07	(0.9-1.1)	0.116
Interaction				
(Aa+aa) X (Bb+bb)	719 (52)	0.51	(0.3-0.6)	2.19E-05
Liklihood ratio test between the models*				
				**P_corrected_**
Corrected				**0.005**

All the models are adjusted for study

* likelihood ratio test comparing Cox' regression models without and with an interaction term.

### Association of the interacting pairs with tumor characteristics

The association between clinical and histopathological features of tumors and genotype combinations of the interacting SNP pairs is summarized in Supplementary Table 6a and b. No significant association was found for the SNP pair from the recessive model. The interacting SNP pair found with the dominant model associated with nodal status (N), with a borderline significant p value of 0.010, and may also associate with metastasis at diagnosis (M), but the difference was not statistically significant. Carriers of the protective genotype combinations (rs17243893-AG+GG and rs57890595-AC+CC) had also a lower frequency of nodal or distant metastasis.

### Corresponding genes in the NF-κB activating pathway

Of the recessive SNP pair, rs5996080 resides in chromosome 22, in the flanking region (31.5 kb downstream) of the NF-κB pathway gene *BAFFR* (B-cell activating factor receptor, also known as *TNFRSF13C*). rs5996080 is in strong linkage disequilibrium (r^2^ = 1) with 15 SNPs located in *BAFFR*. The other SNP in the pair, rs7973914, is located on chromosome 12, 8kb upstream of the NF-κB pathway gene *TNFR3* (TNFR superfamily member 3, also known as *LTBR*)) and 27kb upstream of *TNFR1* (tumor necrosis factor receptor superfamily, member 1a*,* also known as *TNFRSF1A*). rs7973914 resides in a short haplotype block with very few SNPs.

Of the dominant model SNP pair, rs17243893 is located in chromosome 9, within the intron of NF-κB pathway gene *TRAF2* (TNF receptor associated factor 2). The other SNP in the pair, rs57890595, is located in the chromosome 8, and lies in the intronic region of NF-κB pathway gene *TRAIL-R4* (TNF-related apoptosis ligand receptor 4, also known as *TNFRSF10D*). Other nearby genes include *TNFRSF10A* and *TNFRSF10C*.

### Annotation of established functional elements in the site of interacting pairs based on ENCODE data

To investigate the possible functional role of the interacting SNPs or other SNPs in the surrounding LD regions (r^2^ > 0.2), we utilized the ENCODE-based functional annotations at the Haploreg and RegulomeDB databases in order to determine whether they are located within genomic regulatory elements in human mammary epithelial cells (HMEC).

For the recessive SNP pairs, rs5996080 and rs7973914, 35 of the SNPs in the rs5996080 locus may potentially lead to alteration of regulatory elements as defined by transcription factor binding motifs, histone modifications, DNase sites and protein binding regions in HMEC. Of these SNPs, 26 are in strong LD with rs5996080 (r^2^ and/or D’ >0.8). The Haploreg annotation indicated that in HMECs, the majority of rs5996080 proxies are located in regions with enhancer histone marks. We identified 10, 22, and 6 SNPs mapping to regions with promoter histone marks, enhancer histone marks, and DNase hypersensitivity sites, respectively. Of the SNPs mapping to DNase hypersensitivity sites, four were overlapping with regions with promoter histone marks, and three were overlapping with regions with enhancer histone marks (Supplementary Table 7a). According to ENCODE data, rs117492772 (r^2^ = 0.86 with rs5996080) alters a putative transcription factor binding motif of NF-κB. The rs5996080 SNP itself changes the putative motif binding of ERα-a. As for rs7973914, four SNPs in the LD region map to regulatory regions with enhancer histone marks in HMEC. Of these, three SNPs are in strong LD with rs7973914 (r^2^ and/or D’ >0.8) (Supplementary Table 7b).

For the dominant model SNPs, rs17243893 and rs57890595, *in silico* analysis was limited by the short/undefined haplotypes, as indicated above. Haploreg and RegulomeDB annotation maps two SNPs in the rs17243893 locus (rs17243893 and rs35253986, r^2^ = 1 and 0.27, respectively) in HMEC to regions with enhancer histone mark and DNase hypersensitivity sites. (Supplementary Table 7c). No SNPs mapped to HMEC regulatory elements at the rs57890595 locus.

### Association of the interacting SNPs and the expression levels of nearby NF-κB activating pathway genes

To further evaluate whether the interacting pair SNPs might have a functional effect or be related to other functional SNPs that affect the expression level of their corresponding genes in NF-κB activating pathway, we performed eQTL analysis using two publicly available data sets consisting of genotype and gene expression data: TCGA and METABRIC. To this end, we analyzed all available SNPs in the LD region (r^2^ >0.1). The interacting SNPs themselves were not genotyped in either of the data sets, but are represented through LD.

For the recessive model SNP pair, an rs5996080 proxy (rs9620000, r^2^ = 1) consistently associated with higher expression of *BAFFR* in both the TCGA tumor (*P* = 0.049) and METABRIC data sets (*P* = 0.003). We also detected one rs5996080 proxy (rs17002737, r^2^ = 0.79) which associated with the expression of *TNFR1* (P = 0.049), and several SNPs in the locus that correlated positively with the expression of *TNFR3* (rs2269658: r^2^ = 0.5, D’ = 0.8, *p* = 0.00006; rs9620000: r^2^ = 1, *p* = 0.003; rs5996088: r^2^ = 1, *p* = 0.002; rs1023497: r^2^ = 0.4, D’ = 1, *p* = 0.01; and rs133367 r^2^ = 0.2, D’ = 1, *p* = 0.04). There were only four proxies of rs7973914 available in the TCGA/METABRIC data, and none of them associated significantly with the expression level of either *TNFR1/3* or *BAFFR*.

For the dominant model SNP pair, rs17243893 and rs57890595, we did not find any significant correlation between the expression of the few rs17243893 proxies and TRAF2, nor with TRAIL-R4. We identified one SNP rs57890595 in the TCGA tumor data (rs12546238, r^2^ = 0.2), and one (rs4278155, r^2^ = 0.2) in the normal tissue data (*n* = 85), that correlated with the expression of *TRAIL-R4* (*P* = 0.004). Another SNP in the region rs57890595 (rs4871880, r^2^ = 0.1) associated with the expression level of *TRAF2* (*P* = 0.0006) in the METABRIC data.

## DISCUSSION

The postulated association of the NF-κB pathway with tumor progression and patient survival could be affected by interactions of multiple loci, in addition to single locus effects. Here, we aimed to highlight plausible epistatic/non-additive interactive effects of SNPs near relevant genes for further validation and functional analyses. We explored the NF-κB pathway by assessing two-way interactions of 917 SNPs in 75 genes within the NF-κB activating pathway and found evidence of interaction between two pairs of SNPs corresponding to five genes: rs5996080 and rs7973914 by the recessive model, and rs17243893 and rs57890595 by the dominant model. We discovered these associations through a semi-parametric approach, with large sample size, and stringent p value and Hazard ratio criteria determined by power analysis for each model of inheritance. None of the SNPs identified here exerted statistically significant survival effects individually.

Using the recessive model, we identified an interaction between rs5996080, near the gene *BAFFR,* and rs7973914, located in the proximity of *TNFR1* and *TNFR3*. While the SNPs in either of the two loci did not individually associate with survival, they appear to have an interactive prognostic effect: compared to carriers of at least one common allele, the patients carrying the homozygous rare allele of both SNPs had worse 10-year overall survival. We did not observe any statistically significant association between the interacting genotype combination and the clinical and pathological characteristics of the tumors, although it must be noted that we had low power to detect such an association in the first place due to the limited number of cases in the interacting genotype category.

**Figure 3:**
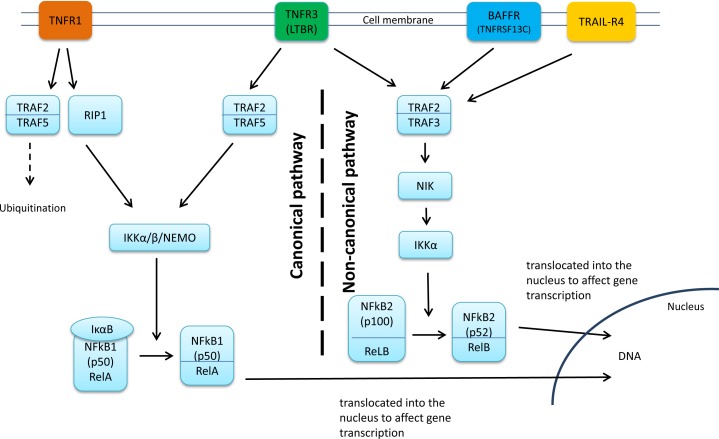
Genes annotated to the SNP pair significant in the recessive model BAFFR activates the NF-κB, mainly through the non-canonical pathway whereas TNFR1 activates the canonical NF-κB pathway and TNFR3 activates both pathways. The survival effect observed in the SNP interaction analyses might be due to simultaneous perturbation of BAFFR and TNFR1/TNFR3 which might affect both the canonical and the non-canonical NF-κB pathways or their dynamics. Genes annotated to the SNP pair significant in the dominant model: TRAF2 functions as a molecular bridge linking the receptors to downstream kinases. TRAIL-R4 has been suggested to activate the non-canonical pathway through TRAF2-NIK-IKK cascade. The observed survival effect by SNP interaction might be mediated through TRAF2 and TRAIL-R4 interplay.

The candidate NF-κB genes (*BAFFR*, *TNFR1* and *TNFR3*) in these loci are in general TNF receptors (Figure 3). Physically, rs5996080 resides in the intron of another gene, *SREBF2* (sterol regulatory element-binding transcription factor-2), and rs7973914 is in the intron of *SCNN1A* (sodium channel non-voltage-gated 1 alpha subunit). *SREBF2* gene is a lipogenesis transcription factor which has been shown to be up-regulated in a breast cancer cell line (HCC1143) compared to normal mammary epithelial cells (MCF10A) [24]. SREBF2 negatively regulates SMAD3 [25] which is demonstrated to physically interact with IKKα (the NF-κB activator). Through this interaction, IKKα is suggested to control the binding of the SMAD complex to DNA and therefore, contribute to the tumor-promoting function of the TGF-beta/SMAD signaling pathway in human MDA-MB-231 breast cancer cell line. However, this process appears to be NF-κB-independent [26, 27]. For *SCNN1A*, a read-through fusion transcript of *SCNN1A*-*TNFR1* has been recently identified in breast cancer cell lines as well as in primary breast cancer tumors, and was not detected in normal tissues [28]. Interestingly, an rs5996080 proxy (rs5996088: r^2^ = 1) associates with decreased expression of *SREBF2* and *SCNN1A* (*p* = 0.014 and *p* = 0.0007 respectively; TCGA dataset only) which might also contribute to the observed interactive survival effect. *BAFFR*, the candidate NF-κB gene in the locus with significant eQTL association with rs5996080, is one of the best known receptors involved in the non-canonical NF-κB pathway and is known to be the physiological signal that promotes the processing of RelB/P100 to ReLB/P52 resulting in the activation of NF-κB2 [29]. Although BAFFR preferentially induces the non-canonical pathway, it has been suggested to activate the canonical pathway as well. However, contrary to its activating role in the non-canonical pathway, the BAFFR impact on the canonical pathway appears minor, and remains ambiguous [30]. TNFR1 signaling, on the other hand, activates the canonical RelA/ NF-κB1 pathway through the induction of the IKK complex, while TNFR3 signaling can activate both the canonical and the non-canonical ReLB/NF-κB2 pathways through the processing of P100 to P52. Perturbations in the function of these genes could therefore be hypothesized to alter the dynamics between pro-apoptotic and anti-apoptotic NF-κB signaling pathways, which in turn may influence cancer progression and outcome, possibly in an epistatic manner. While the biological mechanism behind the SNP-SNP interaction between these loci can only be speculated on at this point, our ENCODE and eQTL analyses indicated that SNPs at both of these loci may influence gene expression, particularly rs5996080, which associates with the higher expression of both *BAFFR* and *TNFR1/TNFR3*. This is in line with the poor breast cancer survival observed in this study, due to the BAFFR-mediated activation of the non-canonical pathway contributing to cell survival [31, 32] and the suggested involvement of the TNFR3 signaling in inflammation-induced carcinogenesis [33]. Indeed, the blockade of the TNFR3 signaling has been used as anti-inflammatory, anti-cancer therapy in some experimental models [34, 35]. Additionally, the ENCODE data indicated that an rs5996080 proxy (rs117492772, r^2^ = 0.86) alters a binding site for the NF-κB transcription factor itself, as well as a putative ERα binding site. In light of this, it would have been interesting to investigate the interaction in subgroups defined by ER status, but such an analysis was unfortunately not feasible due to the limited number of cases in the interacting genotype category. Taken together, we speculate that the combination of the rare homozygous alleles for rs5996080 and rs7973914, or the causative variants in LD with them, might simultaneously compromise the BAFFR and TNFR1/TNFR3 receptors’ function (Figure 3) and breast cancer survival by perturbing both the canonical and the non-canonical NF-κB pathways or their dynamics. However, it is possible that the observed SNP interaction may be due to more complex survival effects by multiple genes in the rs5996080 and rs7973914 loci. This speculation requires further validation, and functional analyses.

Under the dominant model, we identified an interaction between rs17243893 and rs57890595: compared to the patients with wild type homozygous genotypes, patients carrying at least one rare allele for both variants have a better 10-year breast cancer survival. Interestingly, the genotype combination associating with improved survival also tended to associate with negative axillary lymph node status and, although not to a statistically significant degree, less frequent distant metastasis at diagnosis.

rs17243893 is an intronic SNP located in TRAF2, an oncogene which activates NF-κB in epithelial cancers including breast cancer, by activating the NIK-IKK complex (Figure 3). TRAF2 function is best characterized in the TNFR1 signaling pathway [36]. TNFRSF signaling is primarily regulated via TRAFs, and by the activation of NF-κB [37]. The other SNP in this pair, rs57890595, lies in the intronic region of the TRAIL receptor *TRAIL-R4*. Evidence suggests that NF-κB activation by *TRAIL-R4* takes place through a TRAF2-NIK-IKK cascade. This implicates an interplay between TRAF2 and TRAIL-R4 that ultimately promotes cell survival [38]. In the site of rs17243893, our Haploreg analyses show regulatory motif alteration for AP-1: a-c-Fos and c-Jun family member which has been suggested to be reciprocally activated by NF-κB. It has been shown that the inhibition of NF-κB results in the inhibition of *fos* expression and consequently the AP-1 activity in pancreatic adenocarcinoma cell lines (MDApanc-28) [39]. Unfortunately, since neither of the two SNPs in this pair was represented in the TCGA or METABRIC gene expression dataset, we have no compelling evidence that links this pair with the expression of *TRAIL-R4* and *TRAF2*. Nevertheless, these two genes remain plausible candidates for the prognostic interactive effect: TRAF2 /TRAIL-R4-mediated NF-κB activation results in cell survival which would be consistent with the poor prognosis observed in this study under the hypothesis that the rs17243893-rs57890595 (or the causative variants in LD with them) interaction potentiates or otherwise dysregulates this signaling cascade.

In summary, in analyses of 917 SNPs in 75 genes of the NF-κB activating pathway, we identified two SNP-SNP interactions to be associated with survival of the breast cancer patients. We have searched for regulatory elements in these loci and examined their influence on gene expression, and we propose a biological rationale for the observed effects. Yet, one must remain aware of potential data interpretation biases introduced by pathway-based SNP selection. For example, long-distance regulatory effects may extend well beyond the local LD block, and the observed prognostic interactions may therefore be caused by genes other than the primary candidate genes or their neighbors. However, we did not observe significant association between the SNPs in LD with the interacting SNP pairs and the expression level of genes elsewhere in the genome in TCGA and METABRIC dataset consistently. A weakness of our study is that large-scale analyses are potentially at the risk of inflated type I error. As even seemingly significant results can easily be false positives in an underpowered analysis, to address this, we chose to use power calculations to define robust HR thresholds for this study [40]. Nevertheless, in addition to the multiple testing correction methods applied here, further validation of the interaction SNP pairs is required. Moreover, with this approach we were only able to discover interactions with fairly large effect sizes, while any true interactive effects with low hazard ratios would remain undetected. Furthermore, even though the SNPs were selected for their proximity with the NF-κB activating pathway genes and the observed survival effects are consistent with a plausible biological rationale, it is also possible that the observed effect is partially or wholly caused by other nearby genes, especially as the rs5996080 and rs7973914 loci are physically located in introns of other, non-NF-κB, genes. Further functional studies are required to identify the causal elements of the detected effects on patient survival.

The prognosis of complex diseases such as breast cancer is a dynamic process which is influenced by a large number of clinicopathological factors, and is likely also affected by a combination of genetic variants. Compared to an agnostic genome wide analysis, the pathway-oriented approach tested here may increase the chance of obtaining biologically meaningful results with statistical significance regarding SNP-SNP and gene-gene interactions in a prognostic and/or predictive context, although it also necessarily introduces a degree of bias based on the initial hypothesis. These findings can then contribute to functional analyses elucidating the underlying mechanisms of the interplay between genes involved in the NF-κB pathway, and their influence on breast cancer progression and survival.

## MATERIAL AND METHODS

### Ethics statement

Investigation has been conducted in accordance with the ethical standards and according to the Declaration of Helsinki and according to national and international guide. All participants in these studies had provided written consent for the research. All participating studies were approved by the respective ethical review boards and ethics committees: ABCFS (The University of Melbourne Health Sciences Human Ethics Sub-Committee (HESC)), ABCS (Leiden University Medical Center (LUMC) Commissie Medische Ethiek and Protocol Toetsingscommissie van het Nederlands Kanker Instituut/Antoni van Leeuwenhoek Ziekenhuis), BBCC (Friedrich-Alexander-Universitat Erlangen-Nurnberg Medizinische Fakultat Ethik-Commission), BSUCH (Medizinische Fakultat Heidelberg Ethikkommission), CGPS (Kobenhavns Amt den Videnskabsetiske Komite), ESTHER(Ruprecht-Karls-Universitat Medizinische Fakultat Heidelberg Ethikkommission), HEBCS (Helsingin ja uudenmaan sairaanhoitopiiri (Helsinki University Central Hospital Ethics Committee)), KARBAC (Lokala Forskningsetikkommitten Nord), KBCP (Pohjois-Savon Sairraanhoitopiirin Kuntayhtyma Tutkimuseettinen Toimikunta), kConFab/AOCS (kConFab: The Queenland Institute of Medical Research Human Research Ethics Committee (QIMR-HREC)), LMBC (Commissie Medische Ethiek van de Universitaire Ziekenhuizen Kuleuven), MARIE (Ruprecht-Karls-Universitat Medizinische Fakultat Heidelberg Ethikkommission), MCBCS (Mayo Clinic IRB), MCCS (The Cancer Council Victoria Human Research Ethics Committee), MEC (University of Southern California Health Sciences Campus IRB), OBCS (Ethical Committee of the Medical Faculty of University of Oulu and Northern Ostrobothnia Hospital District Ethical Committee), OFBCR (Mount Sinai Hospital Research Ethics Board), ORIGO (Medical Ethical Committee and Board of Directors of the Leiden University Medical Center (LUMC)), PBCS (National Institute of Health (NIH) IRB), pKARMA (Regionala Etikprovningsnamnden i Stockholm (Regional Ethical Review Board in Stockholm)), RBCS (Medische Ethische Toetsings Commissie Erasmus Medisch Centrum), SASBAC (Regionala Etikprovningsnamnden i Stockholm (Regional Ethical Review Board in Stockholm)), SBCS (South Sheffield Research Ethics Committee), SEARCH (Multi Centre Research Ethics Committee (MREC)).

### Study subjects

We used primary data from the studies participating in BCAC [23]. All studies had received approval of relevant local human ethical or institutional boards (see Ethics statement). For each study, the minimum number of events (death) required to enter the analyses was 10. A total of 24 studies contributed here, with data on altogether 30,431 invasive breast cancer cases of European ancestry (Supplementary Table 1). The contributing groups provided data on conventional prognostic and predictive markers: age of diagnosis, tumor grade, size, nodal status, metastases at diagnosis, histological type, estrogen receptor, progesterone receptor, HER2 status, and follow-up and vital status [41].

### SNP selection and genotyping

We included 917 SNPs available in the BCAC iCOGS data set [23], residing within or in a 50kb flanking region of 75 candidate genes involved in the activation of the NF-κB pathway identified from KEGG hsa04064 (www.genome.jp/kegg/) (Supplementary Table 8). These 75 candidate genes included ligands and receptors (e.g. TNF, TLR1-4, and TNFRSF10A and B), membrane molecules (e.g. IRAK2), Kinases (e.g. IKBKB), I-kappa-B cascade (e.g. IKBKG, IRAK1 and TLR8), cytoplasmic sequestering/releasing of NF-κB (e.g. NF-κBIs and TNFSFs), transcription factors (e.g. NF-κB1 and RELs); but not the T-cell specific elements nor the downstream targets of NF-κB [24]. All cases were genotyped as previously described using a custom-built Illumina iSelect array [23]. The genotypes were called using Illumina's proprietary GenCall algorithm. Details of the quality control of the genotype data were described previously [23].

### Statistical analysis

We performed the statistical analyses using R environment for statistical computing version 2.15.2 (http://www.r-project.org/). *P*-values for association of the SNPs with tumor characteristics were calculated using Pearson's chi-squared tests. Power analyses for the survival study were performed using the powerSurvEpi package in R. We excluded SNPs with a minor allele frequency (MAF) <1%. The 10-year overall survival was calculated from the time of diagnosis to the date of death due to breast cancer or other reasons (median follow up time 5.6 years), or to the date of the last follow up. To allow for the inclusion of prevalent cases, time at risk was left censored using date of study entry. Survival analyses included Log-rank tests for assessing the statistical significance of differences between Kaplan-Meier curves for survival, and Cox’ regression models for estimating the hazard ratios (HR)s. All Cox’ models were adjusted for study. All p values reported are from two-sided tests. We performed two-way SNP interaction analysis on a total number of 917 SNPs for recessive (AA = 0, Aa = 0, aa = 1) and dominant (AA = 0, Aa = 1, aa = 1) models of inheritance. To determine the association of SNP-SNP interaction with patients’ survival we used the likelihood ratio test to compare multivariate Cox’ regression models of pairs of SNPs without and with an interaction term (SNP1+SNP2 *vs*. SNP1+SNP2+(SNP1*SNP2). For each pair of SNPs the best model was selected based on the likelihood ratio test p value. To adjust for multiple testing error we applied the Benjamini-Hochberg post hoc method, which is also known to be robust against moderate dependency between SNPs, for example linkage disequilibrium [42, 43]. For the interaction pairs to be considered significant, we set two stepwise thresholds. Firstly, the interactive pairs with *p* value< 0.01 after correction were selected. Secondly, for the selected set of interaction pairs to be considered significant, we applied a threshold for the HR based on the power analysis for each model. With a sample size of 30,431 with 3375 events and the average MAF of 23.4%, in the dominant and recessive models we had 80% power to detect interaction terms with HR > = 1.4 (or 1/1.4 = 0.6) and HR of > = 6.2 (or 1/6.2 = 0.16), respectively. In addition, for the SNP pairs with significant interaction survival effects, subgroup analyses were performed to investigate whether the survival effect was differentially affected by tumor/patients characteristics and/or treatment. However, the analyses were limited to the subgroups with 5 or more death events (i.e. by ER status, lymph node status, and chemotherapy treatment).

### Expression quantitative trait loci (eQTL)

In order to analyze the correlation between the loci of interest and the gene expression we utilized the data from The Cancer Genome Atlas (TCGA) and METABRIC project [44, 45]. From TCGA data set, we retrieved both peripheral blood DNA SNP genotype data, and expression data for 913 primary breast tumors. Additionally, of 913 TCGA cases, we also retrieved expression data from 85 healthy breast tissues. The TCGA expression data is from level 4 RNA-Seq (upper quartile normalized RSEM expression estimates). The TCGA matched peripheral blood DNA SNP genotype data is from level 2 Birdseed files (genotyped on Affymetrix Genome-Wide Human SNP Array 6.0 array and processed using Birdseed). The METABRIC raw genotype data (Affymetrix SNP 6.0 platform) was downloaded from European Genome-phenome Archive (cancergenome.nih.gov). The raw genotype data was processed with Affymetrix Genotyping Console Software following the best practices of Affymetrix SNP 6.0 analysis workflow provided by the program provider. The workflow included a quality control step with Contrast QC, a metric that captures the ability of an experiment to resolve SNP signals into three genotype clusters, applying the sample quality threshold of < 0.4 (the Contrast QC is typically greater than 0.4) and genotype calling using Birdseed v2 including the genotypes with call rate of ≥95%. After the QC process 1328 samples with both genotype and expression data from tumorous breast tissue remained in the analysis. The normalization of mRNA expression data (Illumina HT-12 v3 platform) was performed by quantile normalization utilizing single target distribution, described in detail elsewhere [44]. The cis- eQTL analysis was conducted with R-package Matrix eQTL [46] using linear regression and ANOVA models.

### Functional annotation of variants

Linkage disequilibrium and the haplotype blocks in the SNP regions were examined based on r^2^ using SNAP [47]. To annotate the sequences surrounding our SNPs of interest for regulatory elements we searched ENCODE (Encyclopedia Of DNA Elements) data using Haploreg v2 [48] and RegulomeDB [49]. ENCODE project has conducted high throughput functional assays such as Formaldehyde-Assisted Isolation of Regulatory Elements (FAIRE), Chromatin immunoprecipitation (ChIP), and DNaseI hypersensitivity (HS) to evaluate non-coding functional sequences and regulatory elements such as promoters, enhancers and silencers [50].

## SUPPLEMENTARY MATERIAL FIGURE AND TABLES




















