# Supplementary material for: SNP-SNP interaction analysis of NF-κB signaling pathway on breast cancer survival
Source: Oncotarget. 2015 Jul 22;6(35):37979–94. doi: 10.18632/oncotarget.4991 (PMC4741978; doi:10.18632/oncotarget.4991)
Supplement: Supplementary file 1 [file oncotarget-06-37979-s001.pdf]

SNP-SNP interaction analysis of NF-κB signaling pathway on breast cancer survival

Supplementary Material

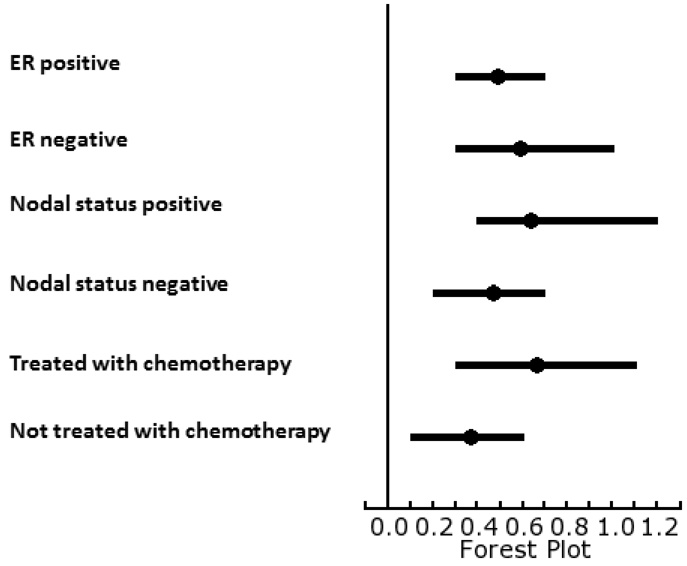

Supplementary Figure 1.

Subgroup analyses: The effect of the interaction pair on patient survival was not confined to any specific subset of patients, i.e.ER (estrogen receptor) positive or. negative, lymph node positive or. negative, chemotherapy-treated or non-treated patients).

Supplementary Table 1. Description of the 24 studies: ascertainment, case definition, and number of patients

Supplementary Table 2. Univariate Cox' regression analyses by 917 studied SNPs for 10-year overall survival.

Supplementary Table 3.

Supplementary table 3. a) The likelihood-ratio test comparing Cox' regression models without and with an interaction term for SNPs nearby or in LD with the interacting-SNPs (rs5996080 and rs7973914, by the recessive model); b) chromosomal distance and LD between other nearby SNPs and rs5996080; c) chromosomal distance and LD between other nearby SNPs and rs7973914

Supplementary Table 4.

Supplementary table 4. a) The likelihood-ratio test comparing Cox' regression models without and with an interaction term for SNPs nearby or in LD with the interacting-SNPs (rs57890595 and rs17243893, under the dominant model); b) chromosomal distance and LD between other nearby SNPs and rs57890595; c) chromosomal distance and LD between other nearby SNPs and rs17243893

Supplementary Table 5.

Subgroup analyses: Multivariate Cox' regression models to assess the interaction between rs17243893 and rs57890595 by dominant model of inheritance. The cases have been subgrouped according to Estrogen receptor status: a) Patients with estrogen receptor positive tumors, b) Patients with estrogen receptor negative tumors, and according to Nodal status : a) Patients with positive nodal status, b) Patients with negative nodal status, and according to treatment : a) Patients were treated with chemotherapy, b) Patients were not treated without chemotherapy.

Supplementary Table 6a.

Association of rs5996080 (A/G) and rs7973914 (G/A) genotype combination (aa+bb vs. the rest) with the clinical and pathological features of the primary tumors

Supplementary Table 6b.

Association of rs17243893(A/G) and rs57890595 (A/C) genotype combination (Aa+aa+Bb+bb vs. the rest) with the clinical and pathological features of the primary tumors.

Supplementary Table 7 a, b, and c.

Regulatory elements from the ENCODE project overlapping with interacting SNPs or their proxies.

Supplementary Table 8.

The 917 SNPs residing within or in 50kb flanking region of 75 candidate genes involved in the NF-κB activating pathway.
